# Experiment and Mechanism of Ecological Restoration of Water Level Fluctuation Zone Based on Redbed Composite Polymer Materials

**DOI:** 10.3390/polym17030306

**Published:** 2025-01-23

**Authors:** Cuiying Zhou, Qingxiu Zhang, Jin Liao, Haoqiang Lai, Zhen Liu

**Affiliations:** 1 School of Civil and Transportation Engineering, Guangdong University of Technology, Guangzhou 510006, China; zhoucy@mail.sysu.edu.cn; 2 Engineering Research Centre for Major Infrastructures Safety, Sun Yat-sen University, Guangzhou 510275, China; zhangqx33@mail2.sysu.edu.cn (Q.Z.); liaoj37@mail2.sysu.edu.cn (J.L.); laihq3@mail2.sysu.edu.cn (H.L.)

**Keywords:** water level fluctuation zone, redbed composite polymer materials, ecological restoration

## Abstract

The water level fluctuation zone, as a transition zone between water and terrestrial ecosystems, is important in maintaining bank stability and regulating the ecological balance of water. The ecosystem residing within the water level fluctuation zone exhibits pronounced fragility, which has resulted in the recurrent manifestation of soil erosion alongside a myriad of other ecological issues. The existing ecological restoration techniques can solidify the soil and protect the slopes but fail to effectively solve soil erosion, which affects the recovery of vegetation. Therefore, in this study, a vegetation survival test under high water head conditions in the water level fluctuation zone and an ecological restoration modeling study of the fluctuation zone based on redbed composite polymer materials were conducted using redbed composite polymer materials as the soil restoration material. The experimental results showed that there were significant differences in the survival status of different classes of plants under high head stress, with different adaptations to environmental stress. In addition, the redbed composite polymer materials effectively improved the water level fluctuation zone, soil water content, conductivity, pH, and other physicochemical properties and improved the stability of soil aggregates. This study reveals the mechanism of action of the material on the soil of the water level fluctuation zone based on the microstructure of the soil, evaluates the restoration effect of the material, verifies the effectiveness of the redbed composite polymer materials in the restoration of the water level fluctuation zone, and provides an effective method for the ecological restoration of the water level fluctuation zone to solve the problem of soil loss and vegetation restoration.

## 1. Introduction

The water level fluctuation zone, driven by reservoir operational cycles, poses significant challenges for ecological restoration due to drastic environmental transformations [1,2]. Traditional restoration methods struggle in this complex and shifting landscape, leading to unpredictable outcomes and failing to address soil loss effectively. Therefore, novel methodologies to enhance soil’s water, particle, and nutrient retention are crucial.

Researchers have focused on three aspects of water level fluctuation zones: ecological environment, soil dynamics, and vegetation patterns. Studies on the ecological environment have highlighted the adverse effects of fluctuations, laying a theoretical foundation for restoration. Soil research has shown detrimental effects on soil integrity, aggregate stability, and physicochemical properties while also mapping soil pollutants and their causes [3,4,5]. Vegetation studies have explored succession patterns, diversity, and restoration constraints, identifying suitable plant species as key for ecological rejuvenation [6,7,8].

In ecological restoration technology research, scholars have proposed systems tailored for water level fluctuation zones, focusing on engineering techniques, biotechnology advancements, and bioengineering combined technologies [9]. Engineering technology includes effective restoration measures such as composite anchor mat systems, beaded flexible bank protection, and ecological bag slope protection. These techniques combat soil erosion and foster vegetation rejuvenation [10,11]. However, these methods suffer from high costs, low plant survival rates, lack of long-lasting restoration effects, and the need for multiple restorations.

Biotechnology aims to develop composite vegetation systems tailored to the zone’s unique characteristics. Researchers have selected plant species with robust adaptability and swift growth rates, implementing scientific planting strategies to expedite vegetation recovery and stabilize the ecosystem. However, widespread application faces challenges due to the abundance and quality of vegetation and the efficacy of ecological functions [12,13].

To address these limitations, bioengineering combined technology integrates engineering principles with biotechnology, optimizing holistic restoration benefits. Yet, traditional soil amelioration techniques like fertilization and tillage are ineffective due to the infertile and loose soil structure within the zone [14,15].

Recently, materials science advancements have yielded innovative approaches using organic polymer materials due to their bonding capabilities, water retention, plasticity, and degradability. These polymers form a network encapsulating soil particles, altering their surface characteristics and enhancing mechanical properties through hydrogen bonding. High-performance water-absorbent resins with intricate molecular architectures sequester and retain substantial water volumes, ensuring a reliable hydration source for plant growth [16,17,18,19,20,21].

Researchers have also explored composites of organic polymers with mineral materials like bentonite and kaolin, improving soil mechanical stability, infiltration, water retention, and nutrient preservation. These composites exhibit restorative effects, augmenting overall soil health [22,23,24,25].

Based on previous research, this study combines redbed composite polymer materials with soil to form a guest soil substrate, investigating guest soil spraying technology for ecological restoration of water level fluctuation zone slopes. The redbed composite polymer materials is a kind of polymer material modified by redbeds, which is mainly composed of redbeds, polymer adhesive material, and polymer water-retaining material [26]. The guest soil spraying technology uses mechanical equipment to mix plant seeds, organic matter, fertilizers, and other materials in a certain proportion and spray them onto the soil surface through high-pressure [27]. By modeling the zone based on these materials, the study verifies their feasibility and effectiveness, revealing the mechanism of action on soil, with emphasis on solving soil loss issues.

The overall objective of our research in this paper is to undertake a comprehensive investigation into the survival potential and morphological adjustment mechanisms of vegetation in reservoir water level fluctuation areas subjected to high water pressure stress. Furthermore, we aim to explore the impact of using red-layered composite polymer materials for ecological restoration in these zones, thereby providing a scientific rationale for ecological restoration and vegetation preservation in such environments. Our study innovatively focuses on:

(1) Evaluating Survival Potential and Morphological Adjustment: By conducting survival experiments under high water pressure conditions in the reservoir water level fluctuation zone, we aim to assess the resilience and morphological adaptability of typical vegetation. Our goal is to identify plant species that exhibit strong resilience in extreme environments, thus providing vital plant resources for ecological restoration efforts. This approach represents a novel contribution to the field by emphasizing the importance of selecting suitable plant species for restoration in challenging conditions.
(2) Modeling Ecological Restoration with Red-Layer Composite Polymer Materials: We propose to conduct pioneering ecological restoration modeling experiments using red-layer composite polymer materials. These experiments will assess the effectiveness of these materials in promoting ecological recovery in reservoir water level fluctuation zones. Additionally, we will analyze the changes in soil physicochemical properties and aggregate content under flood stress, offering new insights into the mechanisms through which these materials can enhance soil stability and promote plant growth. By exploring this uncharted territory, our study seeks to establish a foundation for the innovative use of red-layer composite polymer materials in ecological restoration.

## 2. Research Contents and Methods

### 2.1. Vegetation Survival Experiment Under High Head Conditions in the Water Level Fluctuation Zone

#### 2.1.1. Experimental Purpose

The experimental study aims to evaluate the survival potential and morphological adjustments of typical vegetation in water level fluctuation zones, particularly the low-fluctuation zone, under high water pressure stress. Reservoirs’ operational cycles cause alternating inundation and exposure, posing severe challenges to vegetation. To simulate these extreme conditions and overcome modeling limitations, direct pressure is applied to plants. The survival test assesses resilience and morphological shifts, ultimately guiding the selection of robust plant species for ecological restoration in challenging environments.

#### 2.1.2. Experimental Materials

We have included a more detailed justification for our species choice based on the following principles. The selection of plant species in our study was primarily guided by the unique characteristics of the riparian zone (or water level fluctuation zone) in China, with a focus on the Yangtze River basin, which experiences a subtropical monsoon climate. Within this context, we chose plant species that are commonly used for ecological restoration in this region. These species were selected after considering their typical occurrence and ecological importance in the riparian zone of the Yangtze River. Given the subtropical monsoon climate of the area, these species are well-adapted to the local environmental conditions and have been proven effective in ecological restoration projects. They represent a diverse range of plant types, including herbs, grasses, and shrubs, each with unique tolerance and adaptation strategies to environmental stressors.

**Figure 1:**
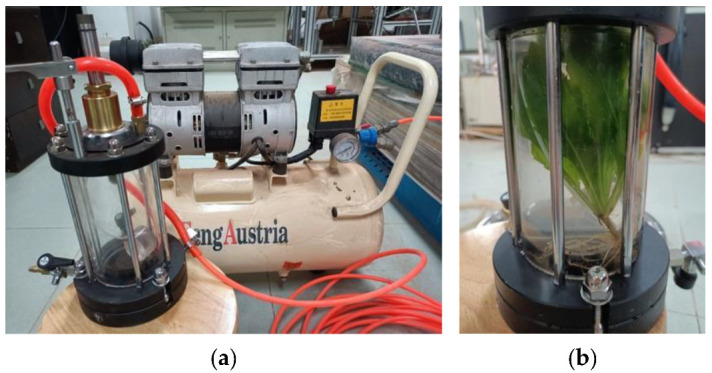
Water pressure test equipment and its connected compressor. (**a**) Water pressure test equipment (made in China, Shanghai); (**b**) Connected compressor.

Specifically, based on the plants of the typical ablation zone in the Yangtze River basin [28], plants subjected to high water head stress used in the test mainly included 12 species of Radix wikstroemiae indicae, Mile-a-minute weed, Perfume flower tree, Cynodon dactylon, Perennial ryegrass, Herba bidentis bipinnatae, Goldthread weed, Lophatherum herb, Candian fleabane, Daun ngokilo, Herb of Japanese Youngia, and Saw grass (Table 1). The equipment used for the test was primarily water pressure test equipment and its connected compressor (see Figure 1).

#### 2.1.3. Experimental Method for Rapid Assessment of the Effects of Fluctuating Water Level Pressure on Long-Term Plant Tolerance

A literature review showed that most reservoir water level fluctuation zones have a water level difference of 0–20 m [29,30,31]. In this experiment, the water pressure strength caused by the 15 m water level difference was taken as the test applied pressure strength. Plants growing in water level fluctuation zones must undergo high water head stress for long periods of time.

In this experiment, to shorten the test time, the creep theory model of fiber materials was used to calculate the water pressure that needs to be applied under a plant stress time of 24 h. The aging theory equation for the creep theory model is as follows [32,33,34]:

(1) $$\varepsilon_{c}=A\sigma^{n}t^{m}$$

where $\varepsilon_{c}$ is the creep strain; σ is the applied stress; *t* is the time; and *A*, *n*, and *m* are the experimental parameters, 0 < *m* < 1. Substituting the water pressure strength of 150 kPa caused by the 15 m water level difference into the model calculations yields that the plant subjected to the water pressure time under 24 h needs to be exerted by the water pressure strength of 300 kPa.

We conducted a comprehensive literature review to identify alternative models used in similar studies to predict plant responses to water stress or stress. We compared the creep theory model with these models (e.g., finite element models, fluid-solid coupling models, and multibody dynamics models) in terms of underlying assumptions, range of applications, prediction accuracy, and computational complexity. Finite element models are computationally intensive and may require longer computational time for large-scale or complex models. Data input is cumbersome, error-prone, and not easily checked and modified. The model setup of the fluid-solid coupling model is complicated and needs to consider the mechanical properties of both the fluid and the solid at the same time. Multi-body dynamics models make it difficult to accurately describe the nonlinear mechanical behavior of plant materials under water pressure. This comparison highlights the unique advantages of our creep theory model, such as its ability to capture the long-term effects of water pressure on plant material properties and its relatively simple mathematical form for ease of implementation.

In order to verify the applicability of the model in plant research, the following experimental steps were designed in this study:

Experimental preparation: representative plant samples were selected, photographed, and recorded before the experiment. Meanwhile, according to the actual water level fluctuation of the reservoir (e.g., 15-m water level difference), the creep theory model was used to calculate the water pressure value (e.g., 300 kPa) that was equivalent to the long-term high water pressure in 24 h.

Experimental setup: Construct a pressurized chamber and ensure that it is well sealed to maintain constant pressure during the pressurization process.

Pressurization process: The plant samples were placed in the pressurized chamber, and the water pressure was gradually increased to a predetermined value (300 kPa) and maintained at this pressure for 24 h. During this period, the sealing and pressure stability of the pressurization chamber were checked periodically.

Observation and analysis: After the pressurization, the water and gas in the pressurized chamber were released, and the plant samples were taken out and photographed again. By comparing the survival status of plant samples before and after pressurization, changes in the root system, stem, and leaves, as well as the tolerance of plants to high water pressure, were assessed.

Model validation: Combined with the experimental data, the accuracy and reliability of the creep theory model in predicting the response of plants to high water pressure in the short term were verified.

Through the above experimental steps and model application, this study aims to deeply investigate the tolerance mechanism of plants to high water stress in the water level fluctuation area of the reservoir and to provide a scientific basis for ecological restoration and vegetation protection.

### 2.2. Model Experiment on Ecological Restoration of Water Level Fluctuation Zone Based on Redbed Composite Polymer Materials

#### 2.2.1. Experimental Purpose

This study employed guest soil spraying on a slope model to assess the efficacy of redbed composite polymer materials in restoring water level fluctuation zones. The materials were integrated with soil to form a guest soil substrate, and the analysis focused on changes in soil physicochemical properties and aggregate content under flooding stress.

#### 2.2.2. Experimental Materials

**Figure 2:**
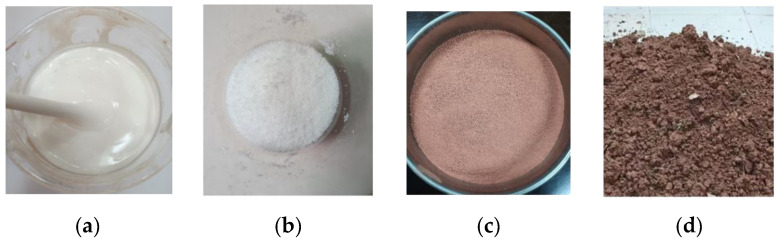
Experimental materials. (**a**) Redbed composite polymer adhesive material; (**b**) Redbed composite polymer water retaining material; (**c**) Redbed weathered soil; (**d**) Guest soil sample.

The test utilized a redbed composite polymer adhesive (see Figure 2a) and a water-retaining material (see Figure 2b). Red-weathered soil (from South China, see Figure 2c) and yellow clay guest soil (typical of South China, see Figure 2d) were used. Soil properties: natural water content 25.26%, air-dried 3.59%, natural density 1.52 g/cm^3^, dry 1.21 g/cm^3^, liquid limit 54.1%, plastic limit 36.1% [35,36].

The water-retaining material used in the experiment primarily consists of sodium polyacrylate, which appears as white particles with a particle size of ≤0.02 mm in a dry state at room temperature. Upon reaching saturation by absorbing water, it transforms into a transparent hydrogel with a water absorption rate as high as 250%. It expands when absorbing water and contracts when releasing it. Within soil, this water-retaining material can efficiently conserve soil moisture for vegetation root growth. During the process of water absorption and release, the volume expansion and contraction of the water-retaining material loosen the soil, effectively improving soil structure and promoting vegetation root growth [24].

The adhesive material is mainly composed of modified polyvinyl acetate, which exhibits a white latex form at room temperature and possesses good dispersibility in water, allowing for preparation as an aqueous solution for application. The adhesive material is capable of self-degrading under natural environmental conditions, ultimately decomposing into CO_2_ and H_2_O. When adhesive material is formulated into an aqueous solution and applied to soil, it can efficiently adsorb and aggregate soil particles, forming soil aggregate structures. This improves soil structure, enhances soil resistance to erosion, ameliorates soil properties, and facilitates rapid vegetation growth [24].

In order to ensure uniformity in the application of redbed composite polymer materials, we followed strict mixing protocols throughout the experiments. The materials were mixed in predetermined ratios based on the requirements of the specific experiment. These ratios were carefully calculated and documented to ensure consistency across all replicates. To further ensure uniformity, we used mechanical mixers to blend the materials thoroughly and regularly checked the mixture for consistency. Additionally, we took samples from different parts of the mixture to verify that the mixing was uniform and that the predetermined ratios were maintained. These measures helped us to achieve consistent results and increase the reliability of our findings.

**Figure 3:**
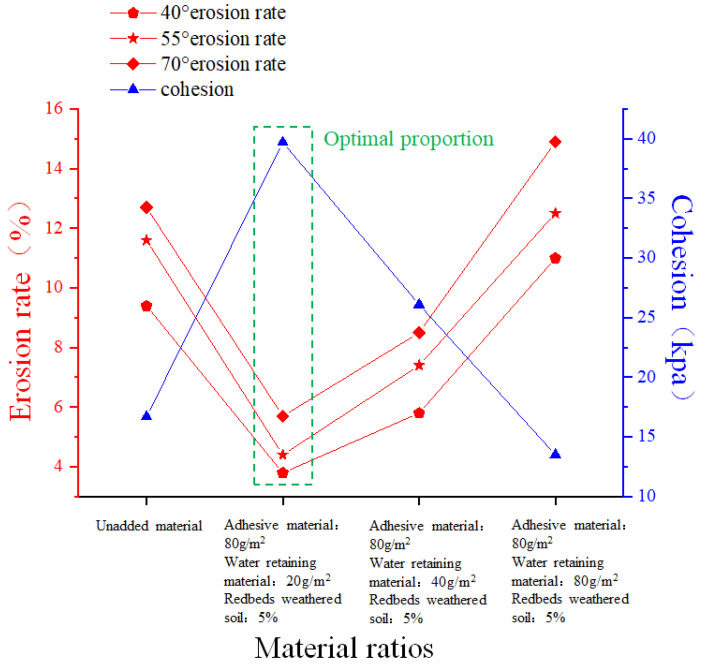
Changes in soil properties with different material ratios.

As can be seen from Figure 3, the slope gradient (40°, 55°, and 70°) is a key topographic factor influencing erosion in water level fluctuation zones. Based on preliminary experiments, an optimal mix of redbed composite polymer materials (80 g/m^2^ adhesive, 20 g/m^2^ water-retaining material, and 5% weathered soil) was formulated. Guest soil samples were processed, and the required materials were weighed. The water-retaining material and weathered soil were mixed into the soil, followed by the adhesive dispersion solution, to create the guest soil base material for slope restoration.

#### 2.2.3. Methods of Studying Slope and Cover Effects on Plant Growth and Soil Properties

**Figure 4:**
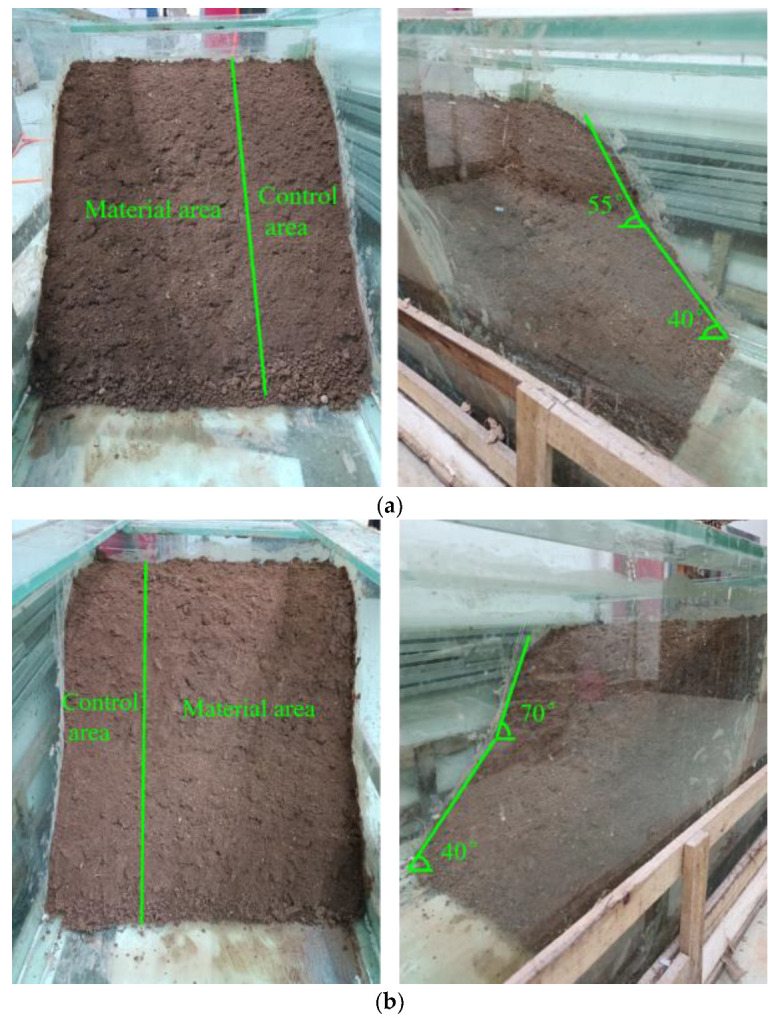
Water level fluctuation zone guest soil restoration of modeled slopes. (**a**) Zone I; (**b**) Zone II.

Two 50 × 50 × 60 cm slopes with 40° lower and 55°/70° upper sections were built from crushed stone. Zones Ⅰ (40–55°) and II (40–70°) modeled water level fluctuation zones. Barbed wire was hung on slopes, and a 10 cm thick layer of redbed composite polymer guest soil substrate was evenly applied. Control, Zone Ⅰ, and Zone II areas were established. *Cynodon dactylon* (L.) Pers. and *Lolium perenne* L. seeds were sown, covered with thin soil, and regularly watered for maintenance (see Figure 4). The degree of soil erosion is mainly assessed according to the soil erosion classification and grading standard SL190-2007 [37] (based on slope and cover).

**Figure 5:**
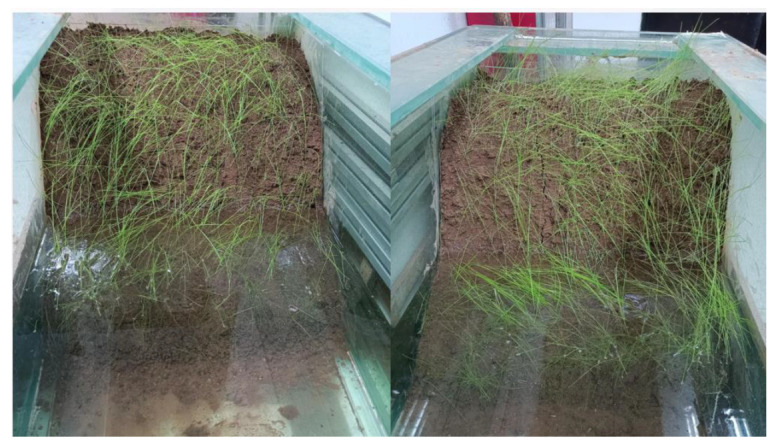
Modeled slopes in flooded conditions.

After 30 days, plants grew to 15 cm, achieving >80% coverage and initiating water storage. Water injection ceased when reaching slope junctures in Zones I & II, flooding the lower slopes (see Figure 5). Soil temperature, conductivity, pH, and aggregate content were monitored every 5 days across Zones I, II, and control. Over 30 days, flooding stress effects on soil physicochemical properties were analyzed. Finally, we performed scanning electron microscopy (SEM) tests on the soil.

## 3. Results

### 3.1. Vegetation Survival Under High Head Conditions in the Water Level Fluctuation Zone

**Figure 6:**
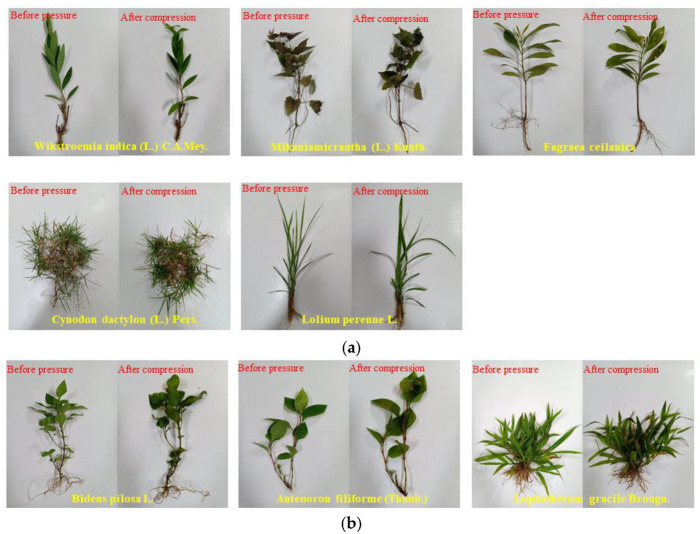

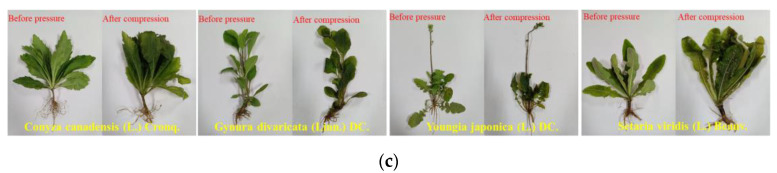
Morphological changes in plants subjected to water stress. (**a**) Morphological changes in surviving plants after exposure to water stress; (**b**) Morphological changes in surviving plants after exposure to water stress; (**c**) Morphological changes in essentially dead plants after exposure to water stress.

Based on plant morphology post-water pressure (300 kPa) (see Figure 6), leaves blackened, indicating death. Survival states were classified as survival, recoverable, or death. Five species (*Wikstroemia indica* (L.) *C.A.Mey.*, *Mikaniamicrantha* (L.) *Kunth.*, *Fagraea ceilanica*, *Cynodon dactylon* (L.) *Pers.*, *Lolium perenne* L.) showed no changes, surviving (see Figure 6a). Three (*Bidens pilosa* L., *Antenoron filiforme (Thunb.)*, *Lophatherum gracile Brongn.*) had slightly blackened leaves but were recoverable (see Figure 6b). Four (*Conyza canadensis* (L.) *Cronq.*, *Gynura divaricata* (Linn.) *DC.*, *Youngia japonica* (L.) *DC.*, *Setaria viridis* (L.) *Beauv.*) showed significant blackening, indicating death (see Figure 6c). Plant survival varied based on their inherent characteristics under high water pressure stress.

The analysis highlights leaves as the most visibly affected plant part under high water stress, crucial for respiration and photosynthesis. Roots and stems remained stable. For water level fluctuation zone restoration, prioritize plants with robust roots, stems, and elongated leaves. This study strengthens our understanding of plant adaptations to high water stress.

### 3.2. Changes in Soil Properties During the Growth Period of a Water Level Fluctuation Zone Model Based on Redbed Composite Polymer Material

The results have been enhanced to provide a more comprehensive comparison between the experimental groups (Zones I & II) and the control groups. This comparison is pivotal in demonstrating the efficacy of redbed composite polymer materials in enhancing vegetation growth and improving soil properties.

**Figure 7:**
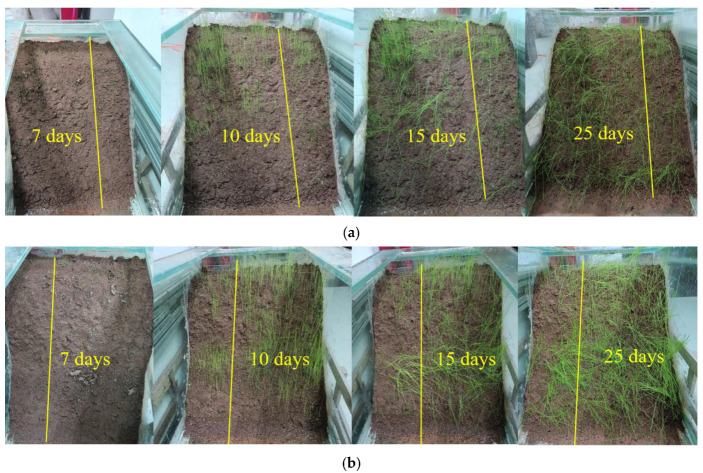
Changes in plant growth. (**a**) Zone I; (**b**) Zone II.

Specifically, the slope of the fluctuation zone maintained with regular watering for 30 days exhibited approximately 15 cm of growth and over 80% coverage when redbed composite polymer materials were used (see Figure 7). Growth charts clearly illustrate earlier germination and accelerated growth in Zones I & II, where the materials were applied, compared to the control areas.

**Figure 8:**
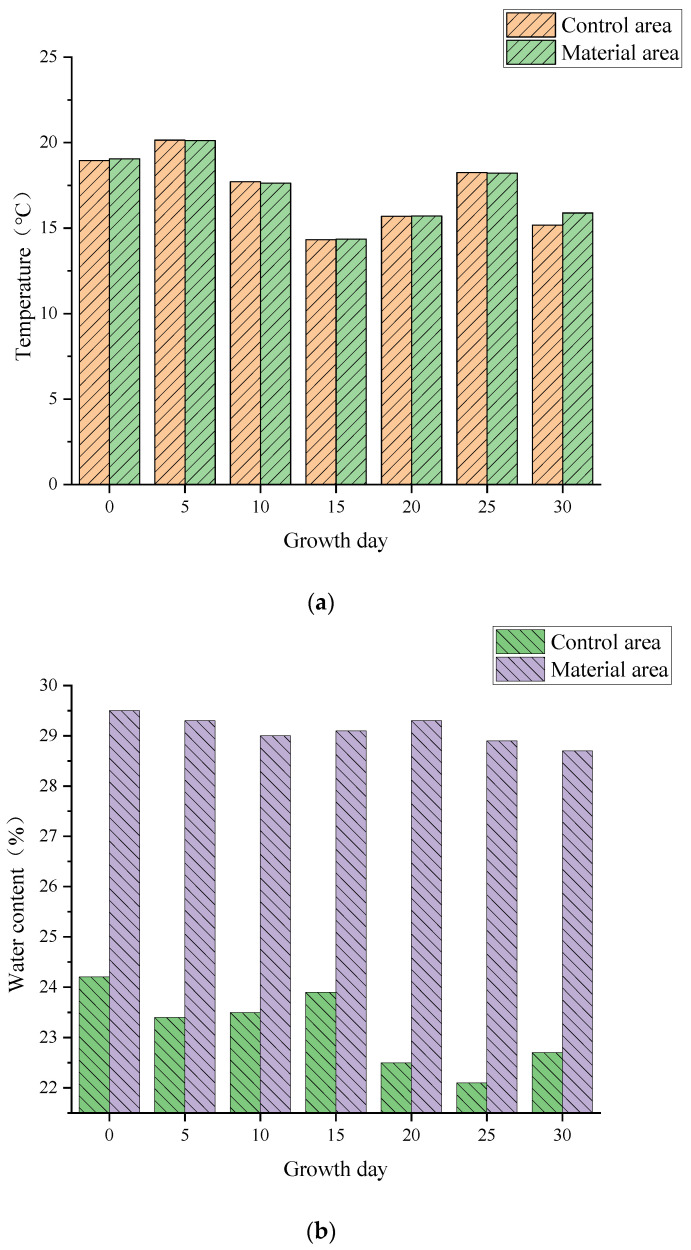

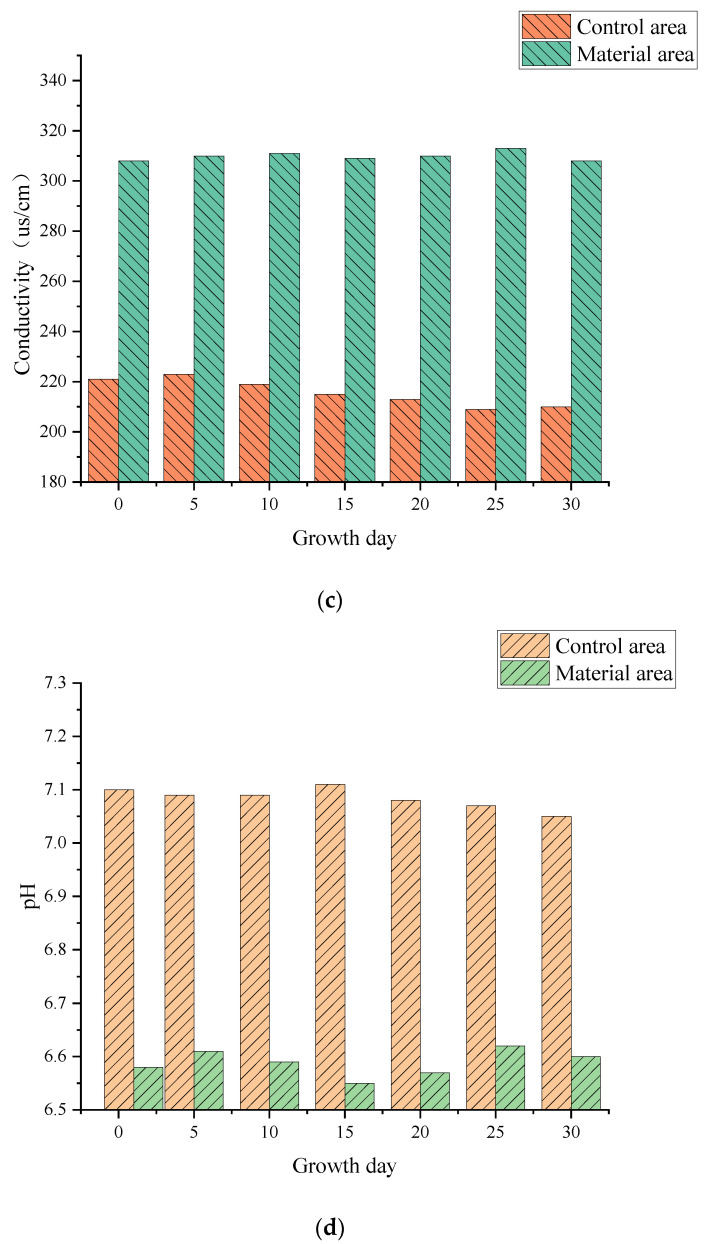

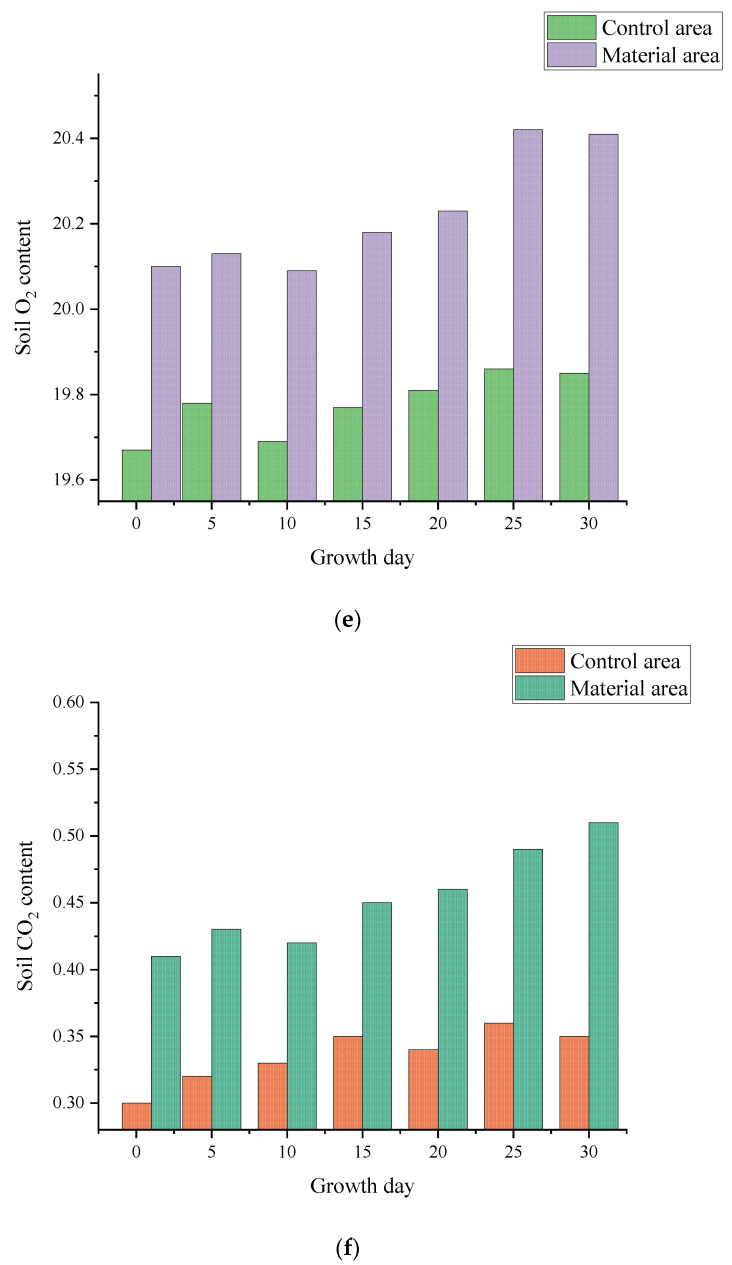
Changes in soil physical and chemical properties. (**a**) Changes in soil temperature; (**b**) Changes in soil moisture content; (**c**) Changes in soil conductivity; (**d**) Changes in soil pH; (**e**) Changes in soil O_2_ content; (**f**) Changes in soil CO_2_ content.

Furthermore, during the plant growth period, soil properties in both the material and control areas of Zones I & II were meticulously monitored every 5 days (Figure 8). While temperature differences remained insignificant, notable variations were observed in conductivity, pH, water content, and O_2_/CO_2_ levels. The material zone soil maintained higher water content and stability, in contrast to the drying trend observed in the control zone soil. Conductivity levels in the material zone remained elevated, in stark contrast to the decline seen in the control areas. The addition of redbed composite polymer materials slightly lowered the pH, but it remained within the neutral range. Most importantly, the material zone soil exhibited significantly higher levels of O_2_ and CO_2_, both of which showed an upward trend as the plants grew.

### 3.3. Changing Law of Soil Properties in Water Level Fluctuation Zone Based on Redbed Composite Polymer Materials Under Water Flooding Stress

#### 3.3.1. Changing Law of Soil Temperature Under Flooding Stress

**Figure 9:**
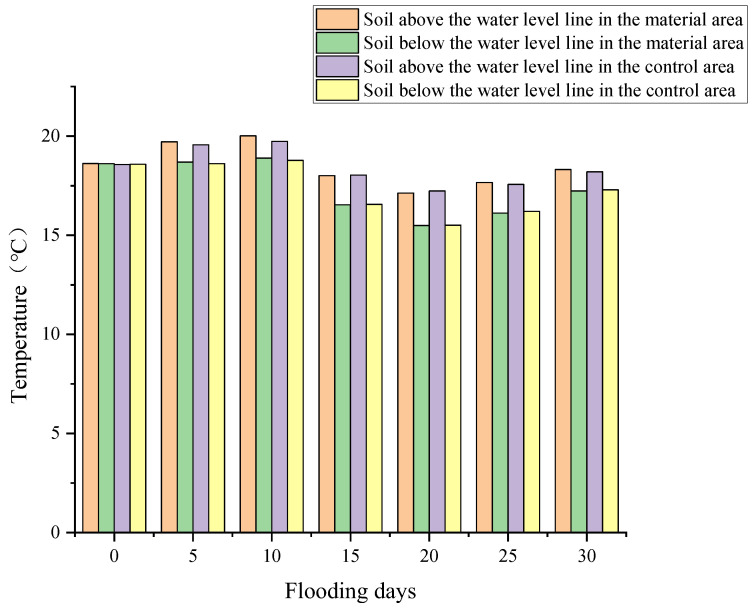
Soil temperature changes under flooding stress.

Soil temperature, crucial for plant growth, varies with flooding (Figure 9). Above the water table, soil temp is higher than below, suggesting flooding reduces temp. However, temp differences between material & control areas remain insignificant, indicating redbed polymer addition does not significantly impact soil temperature.

#### 3.3.2. Changing Law of Soil Conductivity Under Flooding Stress

**Figure 10:**
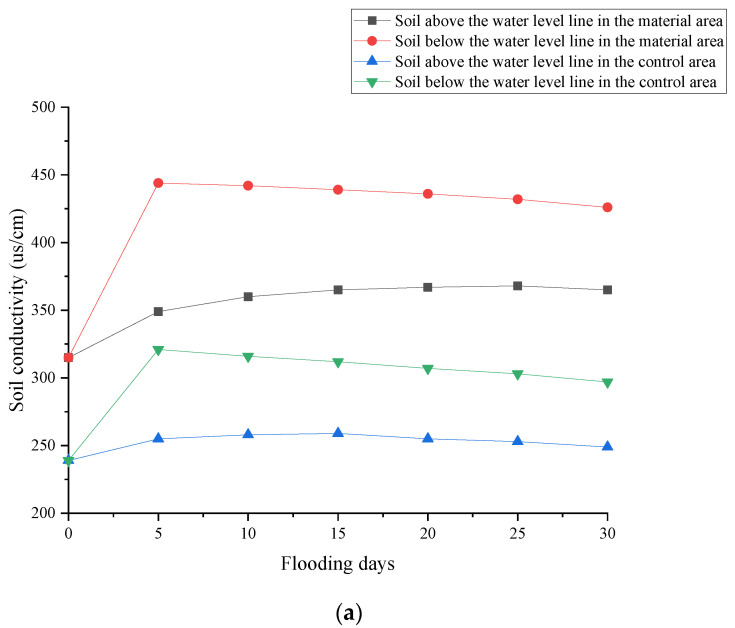

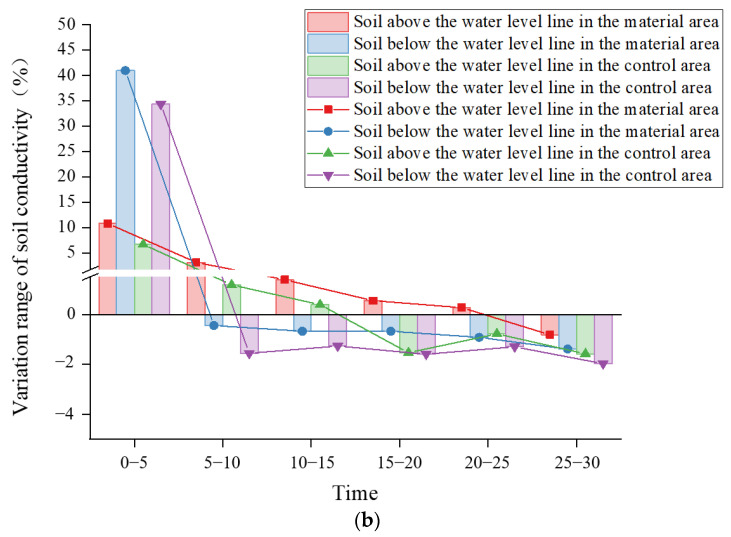
Changes in soil conductivity under flooding stress. (**a**) variations; (**b**) amplitude.

As can be seen from Figure 10a, soil conductivity, crucial for assessing salt impact on plants under water level fluctuations, showed distinct trends. After 5-day flooding, material area soil conductivity jumped 40.9% below the water table, exceeding water-table levels by 27.2%, indicating enhanced water retention. The control area showed milder increases. Over 30 days, material area conductivity fell 4.1%, suggesting salt ion migration, while control dropped 7.5%, reflecting poorer water retention. Upper soil layers saw initial conductivity rise due to moisture migration but decreased later as flooding intensified, revealing dynamic soil-water-salt interactions.

Figure 10b further elucidates soil conductivity changes at 5-day intervals. During the initial 0–5 days of flooding, conductivity increased in all regions, peaking at 40.95% in the material area. From 5–10 days, conductivity declined under flooding stress, with the decline intensifying over time. After 25–30 days, the control area experienced a maximum decrease of only 1.98%. Notably, the decline in conductivity in the material area was less than that in the control area under flooding conditions. In non-flooded soil, conductivity in the material area rose for 25 days, whereas it declined in the control area after 15 days. These comparisons demonstrate the effectiveness of redbed composite polymer materials in retaining soil nutrients and reducing salt ion loss under flooding conditions, which could potentially enhance vegetation survival and improve soil properties.

#### 3.3.3. Changing Law of Soil pH Under Flooding Stress

**Figure 11:**
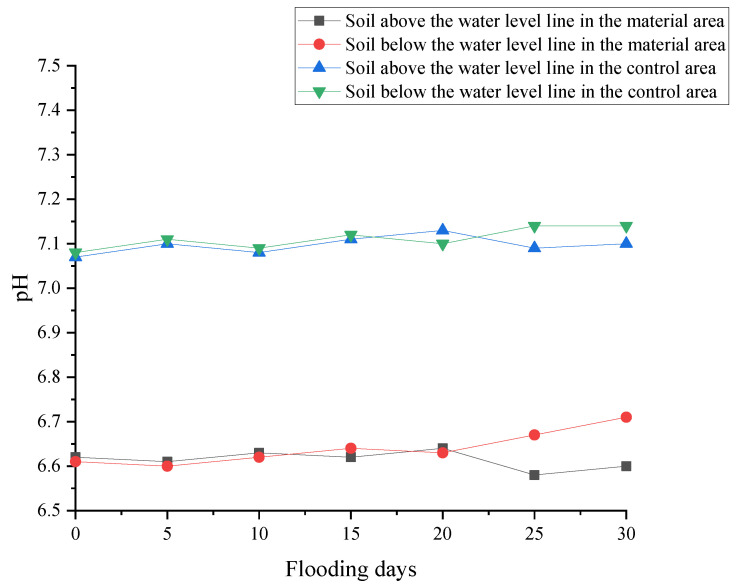
Changes in soil pH under flooding stress.

Figure 11 demonstrates that redbed composite polymer materials significantly decrease soil pH compared to the control group, thereby enhancing nutrient availability. Following a 30-day flooding period, the control soil exhibited an increase in pH, indicative of alkalinity, whereas the treated soil maintained a lower pH level. As the duration of flooding extended, soil pH rose, particularly after 20 days, but this increase was more pronounced in the control group. In contrast, non-flooded soils displayed varied pH levels due to various factors. The materials’ capacity to stabilize soil pH under flooding conditions underscores their potential to bolster vegetation resilience by promoting nutrient retention and recovery.

#### 3.3.4. Changing Law of Soil Aggregates Under Flooding Stress

**Figure 12:**
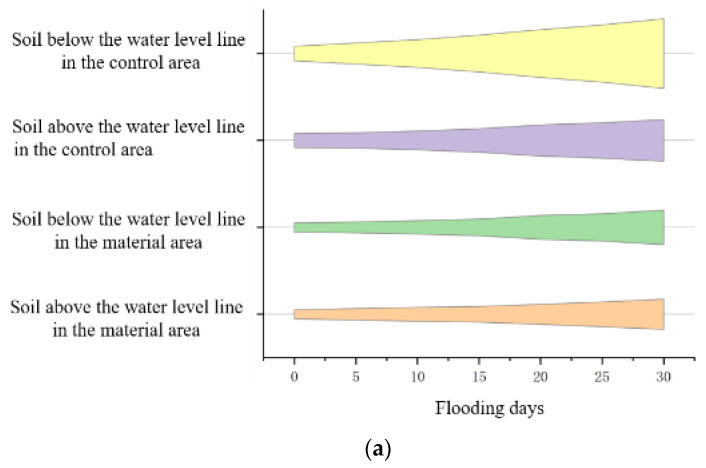

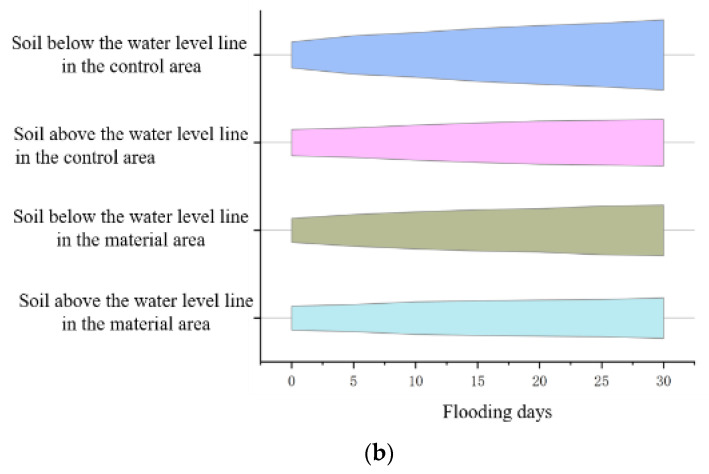
Content of soil aggregates < 0.25 mm in size (in %). (**a**) Soil non-water-stable aggregate; (**b**) Soil water-stable aggregates.

Figure 12 shows redbed polymer materials boost soil aggregate size > 0.25 mm under dry/wet sieving, improving soil structure. Flooding reduces macroaggregate content, more so in water-stable fractions, highlighting hydraulic disruption. Control soil has more fragmented aggregates < 0.25 mm, lacking polymer protection. Higher moisture in a material area is likely due to improved porosity, water retention, and resilient aggregate formation. Materials may also affect microbial activity and organic matter decomposition, influencing water-holding capacity.

### 3.4. Effect of Redbed Composite Polymer Materials on Soil Properties in Water Level Fluctuation Zones

#### 3.4.1. Influence of Redbed Composite Polymer Materials on the Physicochemical Properties of Water Level Fluctuation Zone Soils

**Figure 13:**
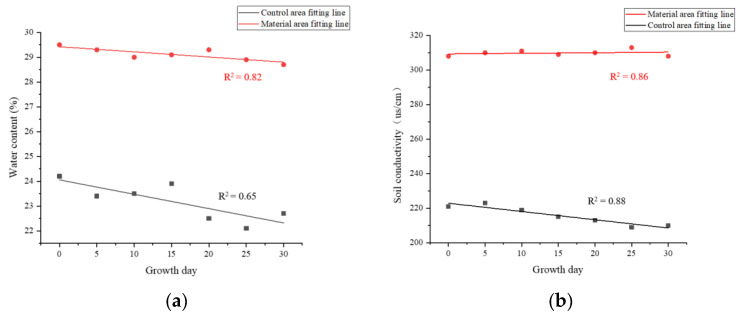

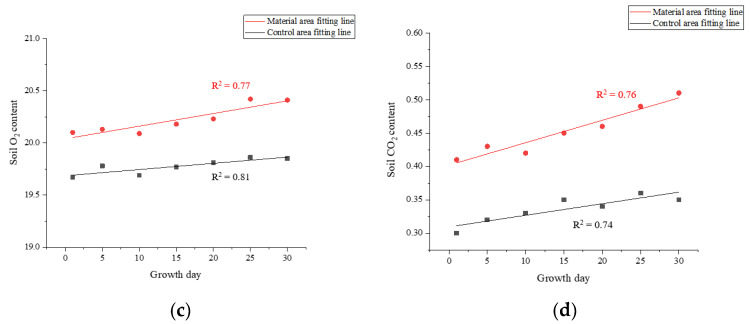
Changes in soil properties over time. (**a**) water content; (**b**) conductivity; (**c**) O_2_; (**d**) CO_2_.

Soil amended with redbed polymer material exhibited a more gradual decrease in water content over time (Figure 13a). After 30 days, its water content remained significantly higher than the control, with minimal fluctuation. In contrast, control soil showed larger fluctuations. This suggests the polymer-amended soil is more stable, resistant to environmental disturbances, and sustains water supply for plant growth.

Soil amended with redbed polymer material showed stable conductivity over 30 days (Figure 13b), while control soil conductivity declined with time. This stability in the amended soil suggests sustained nutrient availability for plant growth.

Soil amended with redbed polymer material showed rising O_2_ and CO_2_ levels over 30 days (Figure 13c,d), faster than the control. This improvement in permeability and air content promotes plant growth and microbial activity.

**Figure 14:**
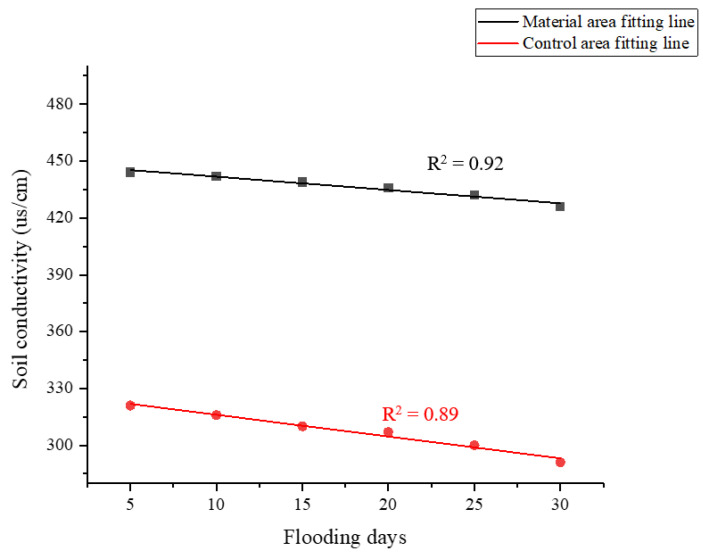
Changing law of soil conductivity with time under flooding stress.

Under flooding stress, soil amended with redbed polymer material showed minimal conductivity decrease (Figure 14), contrasting the control’s significant drop. This stability indicates minimal impact on nutrients, supporting the material’s resilience to flooding.

#### 3.4.2. Influence of Redbed Composite Polymer Materials on Soil Structure in Water Level Fluctuation Zones

**Figure 15:**
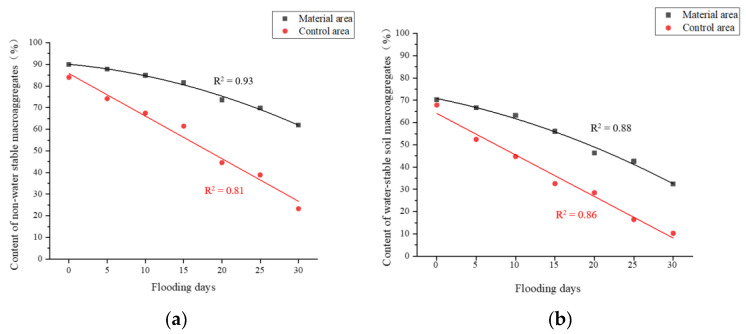
Changing law of soil macroaggregate content with time under flooding stress. (**a**) Non-water-stable soil macroaggregates; (**b**) Non-water-stable soil macroaggregates.

Redbed polymer materials enhance soil structure, which is evident in macroaggregate stability. As flooding time increased, both non-water-stable and water-stable macroaggregates declined, but slower in the amended soil (Figure 15). After 30 days, amended soil retained more macroaggregates, indicating slower erosion and improved stability. Thus, redbed polymers promote soil resilience to flooding and erosion.

The redbed polymer material’s effects on soil diminish over time, but long-lasting benefits remain. It enhances soil structure, fertility, and resilience to erosion and water level fluctuations, aiding vegetation recovery and community stability. This improved soil attracts microorganisms and fauna, boosting biodiversity.

### 3.5. Mechanism of Action of Redbed Composite Polymer Materials on Water Level Fluctuation Zone Soils

#### 3.5.1. Mechanism of Action of Redbed Composite Polymer Bonding Materials on Water Level Fluctuation Zone Soils

**Figure 16:**
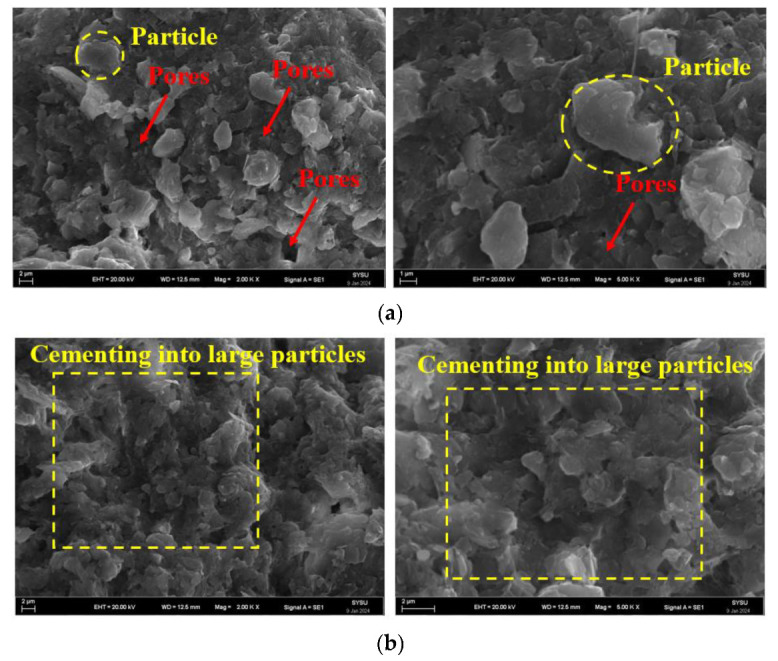
Earthy SEM image. (**a**) Non-water-stable soil macroaggregates; (**b**) Soil samples with redbed composite polymer adhesive materials.

The redbed polymer bonding material wraps fine soil particles into larger aggregates, altering soil mechanics. Upon mixing with water, its macromolecular chains disperse, bonding soil particles via hydroxyl and carboxyl groups, fostering ion exchange and hydrogen bonding [38,39]. This dense packing and material’s diffusion, twisting, and winding in soil form an elastic film around particles, enhancing aggregation and soil stability [40]. A scanning electron microscope (SEM) image of the microstructure of the improved soil sample based on a redbed composite polymer bonding material (see Figure 16). As shown in the figure, under the action of the bonding material, the loose, fine soil particles are tightly bonded to the surface of the larger agglomerates to form larger particles. In this manner, the pores between the soil particles are filled and connected to form a compact whole.

#### 3.5.2. Mechanism of Action of Redbed Composite Polymer Water-Retaining Materials on Water Level Fluctuation Zone Soils

**Figure 17:**
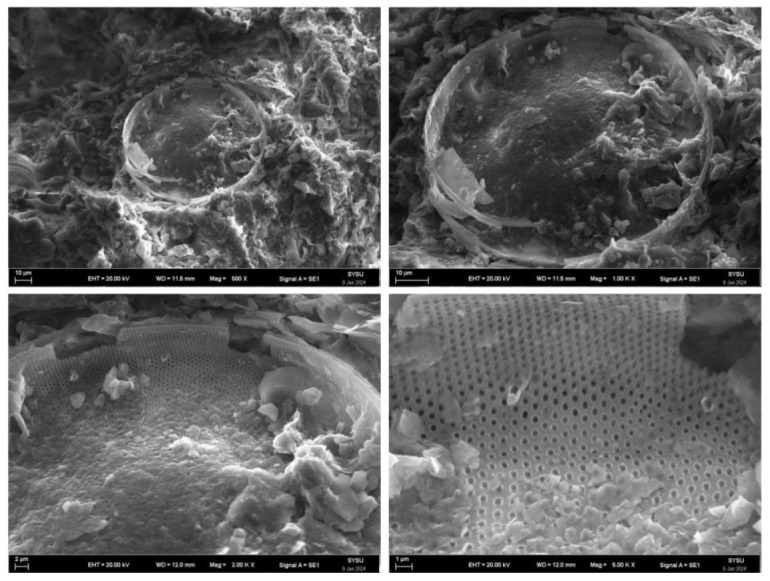
SEM images of yellow clay soil samples based on polymer water-retaining materials.

The redbed composite polymer water-retention material boosts soil’s water absorption and retention thanks to its long-chain polymer and 3D network structure rich in hydrophilic carboxyl and hydroxyl groups (see Figure 17). This microstructure ensures exceptional water-holding capabilities [26]. Upon water contact, hydrophilic groups in the red composite polymer water retention material ionize, turning water into an electrolyte solution. Ion exchange between carboxyl/hydroxyl groups and soil particles fosters solid hydrogen bonds, securing water molecules tightly [35]. Ionization of hydrophilic groups in the redbed polymer water-retainer releases ions, turning water into an electrolyte solution. The resulting potential difference drives water molecules into the material’s interior [41]. Concurrently, ionic repulsion within the material’s network expands its structure, enabling it to accommodate more water molecules due to volume increase.

Upon adding the redbed polymer water retainer to the soil, it absorbs water during saturation, expanding into a hydrogel state. This boosts soil volume and porosity. As soil water evaporates, the material releases water to replenish soil moisture, leveraging its elastic network structure for repeated absorption/release cycles. The material also slows gel-state water evaporation far less than soil, steadily maintaining soil moisture for plant growth.

#### 3.5.3. Mechanism of Action of Redbed Weathered Soil on Water Level Fluctuation Zone Soils

Red-weathered soil enriches the soil with clay minerals (kaolinite, chlorite, and montmorillonite), boosting water retention in fluctuating water levels. Its particles strongly adsorb water, reducing pore water and enhancing soil cohesion, thus improving erosion resistance and strength. Additionally, this layer significantly binds soil and water, enhancing water retention. Clay mineral expansion upon hydration augments soil porosity, enhancing expansion, permeability, aeration, and shrinkage [42,43]. Red-weathered soil enhances soil mechanics and water retention, making it an effective soil-improvement material for ecological restoration.

The elastic membrane of redbed polymer adhesive strengthens soil cohesion, boosting strength and stability. Its water-retaining counterpart absorbs water, expanding to loosen soil and enhance retention. Red-weathered soil, rich in clay minerals, further enriches soil properties and absorption. While these materials significantly improve soil, costs, and longevity are concerns. Incorporating a red-weathered soil layer mitigates costs while sustaining restoration effectiveness.

### 3.6. Evaluation of Ecological Restoration Effect of the Water Level Fluctuation Zone Based on Redbed Composite Polymer Materials

**Figure 18:**
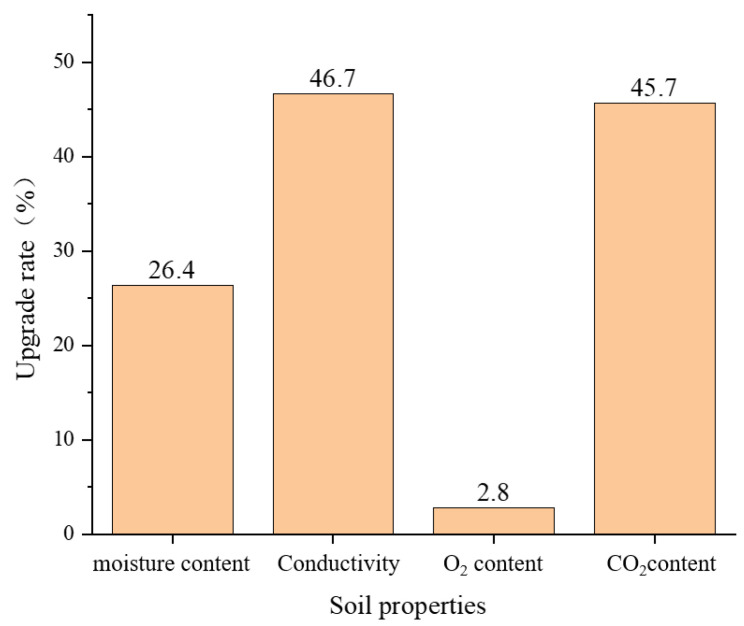
The improvement rate of soil properties in the slope material area of the water level fluctuation zone model compared with the soil properties in the control area.

The model test of redbed polymer material in water-fluctuation zones revealed its profound soil-improvement effects. Figure 18 compares material and control areas after 30 days of plant growth, showcasing marked improvements. Notably, soil conductivity surged by 46.7%, aiding nutrient absorption, while CO_2_ content rose by 45.7%, boosting soil respiration and biological activity. Soil O_2_ increased modestly (2.8%), yet still indicating a positive impact. In summary, redbed polymer materials significantly enhance soil properties, fostering optimal conditions for plant growth.

**Figure 19:**
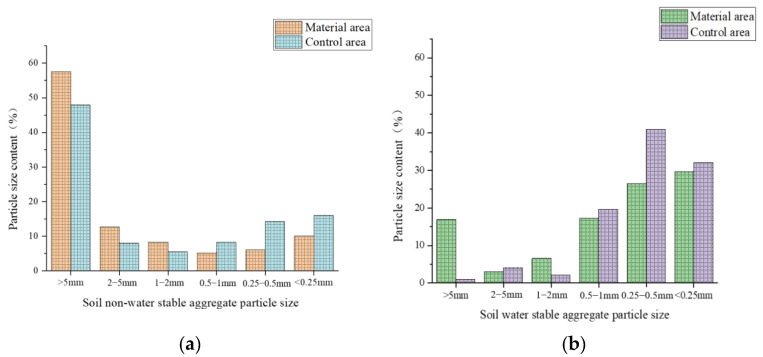
The content of each particle size of soil aggregates in the slope of the water level fluctuation zone model. (**a**) Particle size content of non-water-stable aggregates in soil; (**b**) Particle size of water-stable soil aggregates.

The model test evaluated the redbed polymer’s impact on soil aggregate stability. Figure 19 illustrates that both non-water and water-stable aggregate content increased in the material area versus controls after 30 days. Notably, the >5 mm water-stable aggregates notably surged, altering aggregate size distribution. This enhancement indicates the material’s efficacy in bolstering soil aggregate stability by fostering larger aggregate formation.

**Figure 20:**
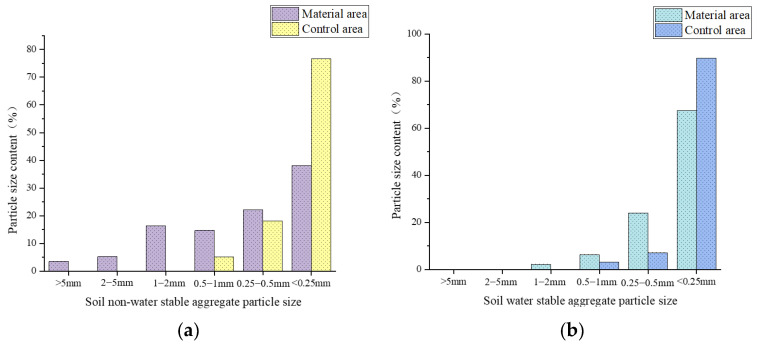
After 30 days of flooding stress, the particle size content of soil aggregates in the model slope of the water level fluctuation zone was measured. (**a**) Particle size content of non-water-stable aggregates in soil; (**b**) Particle size content of water-stable soil aggregates.

The flooding stress test assessed the redbed polymer’s effect on soil aggregate stability. Figure 20 reveals higher > 0.25 mm non-water and water-stable aggregate content in the material area vs. controls after 30 days of flooding. Notably, >5 mm non-water-stable aggregates are also present in the material area. This underscores the material’s efficacy in enhancing aggregate stability under flooding, mitigating soil erosion, and preserving soil integrity.

The redbed polymer material markedly improved soil properties in the water level fluctuation zone, optimizing conductivity, gas & water content, fostering plant growth. It altered aggregate size distribution, boosting large aggregate content, stabilizing soil structure, and resisting erosion under flooding. This material offers a novel, effective approach to ecological restoration in such zones.

## 4. Discussion

### 4.1. Mechanisms of Interaction Between Redbed Composite Polymeric Materials and Soil

#### 4.1.1. Interactions at the Biochemical Level

Soil nutrients and microbial activity: Redbed composite polymeric materials may influence soil microbial colonization and activity through their surface properties. These materials may provide surfaces for microbial attachment, thereby enhancing soil biodiversity and facilitating nutrient cycling.

Organic matter decomposition: The polymer components of the materials may interact with soil organic matter and influence its rate of decomposition. This interaction may help stabilize the organic matter and reduce nutrient losses due to its rapid decomposition.

Plant root growth: By improving soil structure and moisture conditions, red-layer composite polymeric materials may indirectly promote plant root growth and development, thereby enhancing nutrient uptake and utilization.

#### 4.1.2. Physical Interactions

Stabilization of soil structure: Redbed composite polymeric materials enhance the stability of soil structure by aggregating fine soil particles into larger aggregates through their bonding action. This stability helps to reduce erosion and improve the soil’s resistance to erosion.

Moisture Retention and Conductivity: The water-holding agent components of the materials have excellent water absorption and retention capabilities, which can significantly increase the water content of the soil. At the same time, these materials may also influence soil conductivity, promoting uniform distribution of nutrients and water in the soil.

Soil pH and Conductivity: Redbed composite polymer materials may affect soil pH and conductivity through their ion exchange capacity or surface charge properties. These changes may positively affect soil nutrient effectiveness and plant growth.

### 4.2. Practical Implications for Ecological Restoration Practices and Policy Decisions

In this section, we explore the practical implications of our findings for ecological restoration practices and policy decisions, focusing on two key aspects. Firstly, the experimental results validate the effectiveness of the materials used in this study both above and below the water surface in water level fluctuation zones. This verification underscores the material’s capability to improve soil conditions and support vegetation growth under both aquatic and terrestrial environments.

Secondly, our study provides valuable insights and serves as a reference for the application of soil-spraying technology in the ecological restoration of water level fluctuation zones. The techniques employed, particularly the use of redbed composite polymer materials, offer a fresh perspective on enhancing restoration processes in such areas. These materials not only stabilize the soil but also promote plant growth, thereby bolstering the resilience of these ecosystems against environmental stressors such as fluctuating water levels and soil erosion.

From a practical standpoint, engineers and ecologists can leverage these findings to develop more effective restoration projects. The integration of redbed composite polymer materials into these projects presents a promising approach to reducing costs and improving restoration efficiency.

Furthermore, our research has policy implications as well. Governments and regulatory bodies can utilize our findings to formulate or revise policies that support the use of innovative materials and techniques in ecological restoration projects within reservoir areas. By providing financial incentives or regulatory support, these policies can encourage the adoption of advanced technologies like redbed composite polymers.

Lastly, our study emphasizes the significance of long-term monitoring and evaluation in assessing the success of ecological restoration initiatives. By tracking changes in soil properties, plant growth, and ecological recovery over time, stakeholders can make informed decisions about future interventions and ensure the sustained health of these critical ecosystems.

In conclusion, the findings of this study have significant implications for both ecological restoration practices and policy decisions. By validating the effectiveness of the materials used and providing a reference for the application of soil-spraying technology, we can enhance the resilience of water level fluctuation zones and promote sustainable development in reservoir areas.

## 5. Conclusions

The study designed and conducted a vegetation survival test under high water head conditions and an ecological restoration model test using redbed composite polymer materials. The results revealed the adaptability of different vegetation characteristics to high water head stress and clarified the influence law and mechanism of redbed composite polymer materials on soil properties in the water level fluctuation zone. Notably, plant leaf changes were most significant under high water head stress, with survival status varying significantly among plant types depending on their characteristics. Furthermore, the redbed composite polymer material effectively improved soil physicochemical properties, enhancing soil aggregate stability, optimizing the soil environment, and promoting vegetation growth. However, a limitation of this study is the relatively short test period, which may not fully capture long-term effects. Future research should focus on long-term monitoring, expanded environmental factor simulations, cost-benefit analysis, and material formulation optimization. Overall, this research provides theoretical support and practical guidance for plant selection and cultivation under high water head conditions and offers new technical support and solutions for the ecological restoration of water level fluctuation zones, potentially reducing costs and improving efficiency. A limitation of this study is the relatively short test period, which may not fully reflect long-term effects. To address this, future research should focus on long-term monitoring to assess the sustained effectiveness and sustainability of the redbed composite polymer material formulations. This would involve setting up a comprehensive monitoring program to track changes in soil properties, plant growth, and ecological recovery over an extended period. Additionally, cost-benefit analysis and optimization of the material formulations should be conducted to better understand and improve their long-term performance and sustainability.

## Figures and Tables

**Table 1:** Plant species and scientific names.

Names	Latin Name
Radix wikstroemiae indicae	*Wikstroemia indica* (L.) *C.A.Mey.*
Mile-a-minute weed	*Mikaniamicrantha* (L.) *Kunth.*
Perfume flower tree	*Fagraea ceilanica*
Bermuda grass	*Cynodon dactylon* (L.) *Pers.*
Perennial ryegrass	*Lolium perenne* L.
Herba bidentis bipinnatae	*Bidens pilosa* L.
Goldthread weed	*Antenoron filiforme (Thunb.)*
Lophatherum herb	*Lophatherum gracile Brongn.*
Candian fleabane	*Conyza canadensis* (L.) *Cronq.*
Daun ngokilo	*Gynura divaricata* (Linn.) *DC.*
Herb of Japanese Youngia	*Youngia japonica* (L.) *DC.*
Saw grass	*Setaria viridis* (L.) *Beauv.*

## Data Availability

The datasets used and/or analyzed during the current study are available from the corresponding author upon reasonable request.
